# The severe traumatic brain injury in Austria: early rehabilitative treatment and outcome

**DOI:** 10.1186/s13032-016-0035-8

**Published:** 2016-03-15

**Authors:** Emanuel Steiner, Monika Murg-Argeny, Heinz Steltzer

**Affiliations:** Department of Anesthesia, Trauma Hospital Vienna South, Kundratstraße 37, Vienna, 1120 Austria; Department of Neuro-Rehabilitation, Rehabilitation Center for traumatic brain injury patients—Vienna-Meidling, Köglergasse 2a, Vienna, 1120 Austria

**Keywords:** Severe traumatic brain injury, Early rehabilitation, Length of stay, Outcome, Quality of life

## Abstract

**Background:**

Severe traumatic brain injury (TBI) is a great economical and logistic problem in the health care system which reduces the quality of life and productivity of the patient. The purpose of this study is to evaluate the outcome of patients after severe brain trauma according to the course of their rehabilitation.

**Methods:**

Patients with TBI were divided into three groups. Group A; after early rehabilitation (*n* = 16), B; following a standard rehabilitation procedure after work accidents (*n* = 34) and C; undergone standard rehabilitation procedure after accidents at home (*n* = 12). Glasgow Coma Scale (GCS), Post traumatic amnesia (PTA) during acute care, Glasgow Outcome Scale Extended (GOSE) and Functional Independence Measurement (FIM) were measured before and after rehabilitation. Long-term outcomes (12 months post injury) were measured with the Community Integration Questionnaire (CIQ).

**Results:**

Group A showed a significantly shorter time span from hospital admission until rehabilitation center admission than B and C (*p* < 0.001). PTA was significantly lower in group B than in group A (*p* = 0.038). GOSE of patients within group C was significantly lower (*p* = 0.004) at hospital discharge. FIM was significantly higher in B (*p* = 0.005) at the time of admission to rehabilitation center. At the time of discharge FIM showed no significant differences between the groups. CIQ showed a trend to improving scores in group A.

**Conclusion:**

Despite the similar level of severity of TBI and outcome prognosis group A showed the best rehabilitation effect and long-term outcome.

## Background

Patients who suffer traumatic brain injury (TBI) need a demanding and expensive care over a long period of time, which includes intensive care management and rehabilitative treatment. In Austria the incidence of traumatic brain injury is estimated in 312/100 000 inhabitants per year. TBI is not only the main cause of death in a population <45 years, [1] but also the main cause of trauma death in Europe [2]. According to Hyder at al [3], approximately 1,000,000 patients per year are diagnosed with TBI in Europe and despite recent advances still have a mortality rate of 40 % [4].

Enhancements in emergency medical aid and intensive care could improve survival rates and its outcomes. However, the increasing survival rate also implies a rise in the number of patients submitted to rehabilitation, which the health care system has to deal with. Besides traffic accidents, which are the main cause of TBI in Austria, falls below 3 meters are the second most common cause [5]. Low-level falls imply a high risk for elderly people and represent a increasing problem with the further ageing of the western civilization. This issue is even more serious in respect to the long and wearing rehabilitation process [6]. Sanchez et al. [7] already reported that falls have become the leading cause of severe TBI in adults, outpacing traffic accidents in Pennsylvanias ageing population.

With the need of a prolonged intensive care treatment, economic costs rise and strain the health care system. This called attention within the public health sector for TBI and its treatments in the last 15 years. However there are no international guidelines for early rehabilitative treatment available yet.

Early rehabilitation is a process which requires various specialists. A team of the intensive medical care unit, neurologists, physiotherapists, logopedics, occupational therapists, psychologists, social workers and nurses work together to achieve the best therapy for each individual patient [8]. The coordination and the time management of the therapy plays an important role for optimal results.

Andelic et al. [9] and Sörbo et al. [10] described the importance of an unbroken chain of rehabilitation for the outcome of severe TBI patients and confirmed the need for an early start of rehabilitative care. Also Kunik et al. [11] investigated the influence of time to rehabilitation admission demonstrating that early admission to rehabilitation results in better functional outcomes and a reduced length of stay in rehabilitation centers. Zampolini et al. [12] emphasize the importance of early rehabilitation interventions, to allow for the best possible outcomes of the patient. Any delayed admission to rehabilitation may influence the rehabilitation process and result in poorer outcomes, the timing of admission to rehab therefore is crucial. Thus, in order to optimize and accelerate the treatment process patients should receive rehabilitative treatment as soon as possible [13]. Additionally, patients should be transferred directly from the intensive care unit to a specialized rehabilitation center when stabilized in respiratory and circulatory function. Also neurosurgical procedures should be finished, with the exception of skull bone implantation.

In Austria workers compensation, the Austrian Workers’ Compensation Board (AUVA), is split from general health insurance and keeps its’ own trauma hospitals and rehabilitation centers. One of these facilities is the trauma hospital Meidling, which has an exceptional position since it is directly next to a rehabilitation center of the insurance company. Because of the close collaboration early rehabilitation and strictly organized transfer is possible with less administrative barriers than in other hospitals and rehabilitation centers. Other patients undergo different administrative processes and therefore suffer from different delays depending on the hospital they are primary admitted and if their accident is listed as workplace accidents or outside work accidents.

The goal of this study was to demonstrate that patients who are admitted to early rehabilitation as soon as possible at the intensive care unit and complete following rehabilitation programs without any delay reach better short and long-term outcomes as well as a reduced length of stay in the facilities.

## Methods

### Subjects

Between 2006 and 2008 this study was conducted by the cooperation of the trauma hospital Meidling (Unfallkrankenhaus Wien Meidling) and the rehabilitation center Meidling (Rehabilitationszentrum Meidling) in Vienna, Austria and composed of a prospective early rehabilitation group and two retrospective control groups with a total number of 62 patients.

The early rehabilitation group, group A, included 16 patients with severe TBI who were admitted directly to the trauma hospital Meidling or were transferred from other trauma centers after consultation. They fulfilled exclusion and inclusion criteria.

According to the Austrian health care system the control groups were defined as follows. Group B contained 34 patients with TBI after accident at work, treated in other hospitals and fulfilling the criteria. Members of group C suffered a TBI from other causes and were treated in other hospitals (*n* = 12). The controls, groups B and C were retrospectively recruited from the rehabilitation center Meidling.

### Exclusion and inclusion criteria

Included were patients 1) with severe traumatic brain injury and a Glasgow Coma Scale (GCS) score ≤8 before intubation and/ or pathological CT findings, 2) with no more than two concomitant injuries, that have no impact on the progress and for the 2 next higher organ systems equivalent an Injury Severity Score (ISS) ≤13 independent of the brain injury’s Abbreviated Injury Scale (AIS, which means that additional only two organ systems are allowed to have an AIS of maximal 1x“3” and 1x“2”), 3) who are haemodynamically stable (even though under catecholamines), 4) whose intracranial pressure (ICP) and cranial perfusion pressure (CPP) are stable (also under therapy), 5) who are between 16 and 70 years old, 6) with possible assessment of findings for prognosis, 7) whose prolonged hospital/rehabilitation center stay was assessable because of the severe injury.

Exclusion criteria were 1) failure to perform inclusion criteria, 2) more than two concomitant injuries with impact on the progress of the patient, that are independent for the brain injury’s AIS with an ISS ≥13, 3) contact for transfer >7 days after trauma, 4) transfer within 14 days after trauma not possible, 5) not curative treated malignant tumor, 6) complete paraplegia, 7) stroke with defaults and/or severe dementia and/or severe degenerative CNS disease, 8) younger than 16 or older than 70 [14].

### Study algorithm

Patients of group A were admitted either immediately after trauma and surgery or after consultation from other hospitals to the intensive care unit (ICU) as soon as possible, if a free bed was available. In the ICU after the acute phase of trauma an interdisciplinary team, consisting of neurologists, intensive care specialists, traumatologists, physiotherapeutics and nurses started with a rehabilitation oriented intensive care treatment comprising basal stimulation including occupational therapy , physiotherapy*,* facio-oral therapy, and speech therapy if possible [8]. Initial rehabilitation therapy was focused on the prevention of secondary injuries like muscle contractions and infections as well as the prevention of complications due to dysphagia.. Goals and rehabilitation regime were establishes on the basis of the AUVAs’ rehabilitation manual [15] and the references described by Barnes [16]. The treatment of the patient was accompanied by the psychosocial mentoring of the family members to provide their positive input and contact to the patient’s rehabilitation process. In case the patient was stable enough, he/she was transferred to the rehabilitation center as soon as possible, to continue with the rehabilitation therapy. The referral was specifically organized to avoid any interruption in the treatment process. In the rehabilitation center the rehabilitation process faced its next phase and therapies were continued with the highest possible intensity. The postacute phase of the treatment consisted of a general rehabilitation program [15]. All three groups were part of the same rehabilitation program, they differed only in the timing of the admission to rehabilitation. Groups B and C started when they arrived at the rehabilitation facility.

### Measured parameters

The following period of times of stay in hospital and rehabilitation center were documented: duration until admission to the rehabilitation center (days to rehab. center), duration of the whole period of rehabilitation (days of rehab.) as well as their stay in the special rehabilitation unit of the rehabilitation center (urgent tract).

The following scores were evaluated: GCS was evaluated prehospitally or in the emergency room. The Glasgow Outcome Scale Extended (GOSE) was recorded before the patient was transferred to the rehabilitation center (group A) or discharged from the ICU, if there was a waiting period for a therapeutic bed in the rehabilitation center (groups B,C), to characterize hospital outcome. The Functional Independence Measurement (FIM) score was used at admission (AFIM) and discharge (DFIM) from rehabilitation center. Also the duration of Post traumatic amnesia (PTA) was recorded. The Community Integration Questionnaire (CIQ) was sent to the patients 12 months after trauma.

Scores of GOSE, CIQ and FIM were categorized in unfavorable (reached points <50 % of possible points), moderate (reached points between 50 % and 75 % of possible points) and favorable (reached >75 % of possible points).

The GCS is an acute trauma score, which is used to evaluate the state of awareness at the time of the accident and to predict possible neurological physical injuries.

PTA represents the severity of the brain injury. In doing so a duration less than 24 hours is comparable to a GCS of 12–15 and correspond to a mild TBI, a duration of 1 to 7 days is comparable with a GCS of 9–11 and is called a moderate TBI and 1–4 weeks equivalent a GCS of 3–8 and speak for a severe TBI. A duration of over 4 weeks implies a very severe TBI. Additionally PTA is the best acute and long-term predictor of the extent of deficits after TBI. It also serves as an indicator for the length of stay and as a predictor for the treatment cost [17–19].

The FIM is a scoring system to measure the severity of limitations, caused by a TBI or other diseases and accidents. It can also be used to review the progress of the patient’s rehabilitation [20].

The CIQ evaluates the reintegration of the patient in the daily life and his capability to cope with work, household, shopping and social life. The score consists out of the three sub-items: home integration, social integration and integration into productive activities. It was especially developed for people who have suffered a TBI [21].

### Ethics

Because of the retrospective- partly prospective epidemiological study design and no additional intervention in the prospective group than the hospital´s normal procedures there was no ethics committee involved. Consent and permission wasn’t obtained since the study was performed in an acute setting with patients in a state of highly decreased consciousness.

#### Statistics

The statistical analysis was performed using SPSS version 22. Comparability of groups and outcome variables were analyzed by one-way ANOVA followed by post-hoc analysis (Mann-Whitney U-Test). A p-value of less than 0.05 was established as the level of significance. Descriptive data is presented with mean and SD.

## Results

There were no significant differences in age and GCS between the groups. Post traumatic amnesia (PTA) showed to be significantly shorter in patients of group B than in A (*p* = 0.038, Table 1). PTA was present in a number of patients after release. Two patients in A, six patients in B and four patients in C suffered from posttraumatic psychosis and permanent disorientation.

**Table 1:** Patients and injury characteristics, clinical features and facility stay

	Group A (*n* = 16)	Group B (*n* = 34)	Group C (*n* = 12)		(*n* = 62^a^)
Parameter		mean ± SD		*p*-value*	n total
Age	37.31 ± 10.92 (*n* = 16)	37.83 ± 13.43 (*n* = 34)	47.56 ± 11.98 (*n* = 10)	0.061	60
GCS	5.07 ± 2.19 (*n* = 15)	5.86 ± 3.35 (*n* = 20)	6.70 ± 3.62 (*n* = 11)	0.568	46
PTA^b^	40.14 ± 26.46 (*n* = 14)	23.07 ± 10.63^c^ (*n* = 28)	45.00 ± 28.65 (*n* = 6)	0.039	48
days to rehab. center	39.31 ± 18.50^d^	81.34 ± 53.42	80.45 ± 33.38	<0.001	62
urgent tract/d	53.13 ± 60.38	27.11 ± 54.94^d^	82.45 ± 57.54	0.001	62
days of rehab.	103.38 ± 49.46	85.74 ± 44.91	104.82 ± 37.64	0.304	62
Total/d	143.25 ± 56.37	158.51 ± 87.01	175.55 ± 50.67	0.325	62
AFIM	89.13 ± 44.99 (*n* = 16)	119.06 ± 37.01^d^ (*n* = 34)	85.60 ± 32.43 (*n* = 11)	0.005	61
DFIM	127.25 ± 32.91 (*n* = 16)	125.54 ± 36.05 (*n* = 34)	122.40 ± 20.90 (*n* = 11)	0.149	61
GOSE	4.94 ± 1.29	5.29 ± 1.34	3.91 ± 1.04^d^	0.004	62

^a^n total of groups

^b^PTA was in 12 cases delimitable and in two cases (both Group C) unknown

^c^significantly different from A

^d^significantly different (post-hoc analysis)

*One-way ANOVA

Group A was earlier admitted to the rehabilitation center than the other groups (Table 1). The duration of the whole period of rehabilitation and the total time of admission to release, including the stay at the hospital and rehabilitation center, was not significantly different. Length of stay in the urgent tract was shorter in group B than in A (*p* = 0.031) and C (*p* = 0.001, Table 1).

GOSE showed significant differences between the groups (Table 1). Group C revealed significant lower values compared to A (*p* = 0.029) and B (*p* = 0.002).

Analysis of AFIM revealed that Group B had significant higher scores than A (*p* = 0.016) and C (*p* = 0.005). The DFIM score exposed no significant differences between groups (Table 1).

Out of 62 CIQ questionnaires 50 % returned, 29 could be analyzed. Of group A 8 patients replied. To establish a more meaningful comparison group the control groups were united (group BC, Table 2, *n* = 21).

**Table 2:** Injury characteristics, clinical features and facility stay of long-term outcome group

	Group A (*n* = 8)	Group BC (*n* = 21)	(*n* = 29^a^)
Parameter		mean ± SD	n total
Age	37.75 ± 10.65	40.48 ± 14.19	29
GCS	5.25 ± 1.75 (*n* = 8)	6.36 ± 3.48 (*n* = 14)	22
PTA^b^	38.38 ± 25.91 (*n* = 8)	26.20 ± 12.51 (*n* = 15)	23
days to rehab. center	40.50 ± 21.74	87.14 ± 48.58	29
urgent tract/d	41.00 ± 59.69	31.00 ± 56.97	29
days of rehab.	110.75 ± 54.10	88.14 ± 48.72	29
Total/d	144.75 ± 50.45	164.67 ± 86.95	29
AFIM	99.88 ± 43.35	112.14 ± 39.14	29
DFIM	139.12 ± 1.81	126.57 ± 30.45	29
GOSE	5.50 ± 1.07	5.29 ± 1.42	29
CIQ	16.47 ± 4.94	14.06 ± 5.47	29

^a^n total of groups

^b^PTA was in 6 cases delimitable

The categorization of the CIQ showed that, 1/8 patients of group A recovered well, 5/8 remained in a state of moderate disability and 2/8 in a state of severe disability whereas in group BC, none of the patients achieved good recovery results, 12 remained in a state of moderate disability and 9 in a state of severe disability.

## Discussion

By means of PTA as a measure, in group A 11 out of 16 patients remained for more than 4 weeks in a state of post traumatic amnesia and could be classified as very severe, whereas in group C 10/12 and in Group B 11/32 fulfilled this definition. These numbers indicate that patients of the groups A and C suffered more severe TBIs than patients of B even though the GCS scores did not reveal any significant differences (Table 1). GCS was chosen as one of two trauma scores as it is quick and easy to handle in order to accomplish a reliable assessment of TBI severity with prognostic value. Second PTA was evaluated to specify trauma severity, predict outcome and reduce possible bias through adulterated GCS scores [9, 19, 22].

The GOSE scores resulted significantly lower values in control group C compared to the other two groups. The outcomes of group C are comparable to the findings of Livingston et al. [6] who described the hospital outcome using the Glasgow Outcome Scale (GOS) as 4 % favorable, 30 % moderate and 66 % unfavorable. These findings suggest, that due to the intervention of an early rehabilitation program, group A has resulted in better outcomes, compared to all the other groups, which have been admitted to rehabilitation later than group A. The fact that, patients of group C had to overcome more difficult administrative burdens, since these TBIs were not categorized as work accidents, should also be taken into consideration. Patients of this population with good clinical preconditions concerning their neurological deficits, are often not admitted to rehabilitation centers of the AUVA but instead may continue the rehabilitation process with private trainers. Therefore mainly patients with inferior remission are permitted to AUVA rehabilitation centers. The better GOSE results of group B could be also explained, by the fact that they might have suffered less severe injuries, compared to the other groups. This view, may be supported by the higher AFIM score of group B and their shorter stay in the urgent care tract of the rehabilitation center (Table 1).

The decreased time until admission to rehabilitation center of group A may negatively influence their AFIM since the patients were transferred in a sub acute setting. In group C low AFIM scores could also represent the selective admission process.

By looking at the achievement of favorable and unfavorable rehabilitation outcome (DFIM), group A showed a favorable outcome on the same level as control group B, though A included more severe injuries, which has been indicated by PTA, and had prolonged stays at the urgent rehabilitation tract. Kunik et al. [11] demonstrated the negative impact of delay of rehabilitation center admission using FIM as an outcome parameter. It shows lower admission and discharge FIM scores (AFIM total: 54.33 ± 22.09, DFIM total: 95.83 ± 14.30) than our group of patients, however the severity of injury was not assessed by the study of Kunik et al.. DFIM Scores of all groups were similar at discharge, however it should be emphasized that group A achieved the best rehabilitation outcome, even better outcomes than group B. Also the DFIM of group C did not significantly differ from the other groups but this control group displayed the lowest score and in all time related parameters, the longest stay.

Another finding of our study was that group A showed a better long-term outcome measured by CIQ than the control group BC (Table 2) Therefore we conclude that group A has obtained an ideal management to reach optimal levels of rehabilitation progress.

Other studies verified already positive effects of early rehabilitation. Barnes [16] illustrated the benefits of “post acute rehabilitation” unrolling papers on the subject of the last century. Godbolt et al. [23] described the negative association of delayed rehabilitation admission and outcome just recently and suggested that measures to ensure timely rehabilitation admission may improve outcome. Andelic et al. [9] described a favorable long-term outcome using GOSE of 71 % in the early rehabilitation group of their study. In contrast the study’s control group only achieved 37 %. In addition other studies from Mammi et al. [13], Sörbo et al. [10] and Kunik et al. [11] show similar findings. Their results also indicate that due to an earlier start of the rehabilitation project, reviewed patients show shorter stays in hospital and rehabilitation facilities. These outcomes couldn’t be confirmed totally by our study. Unlikely duration in the rehabilitation center showed to be shortest in control group B. These results could be explained by the more severe TBIs of A and the low GOSE scores of C, which lead to longer stays in the rehabilitation center. The total duration of the treatment period didn’t differ significantly, nonetheless A showed shorter total stays than B and C (Table 1).

Horn et al. [24] investigated the relation between patients who had suffered a stroke and early rehabilitation on 830 participants and concluded that especially severe injured patients may benefit from an early and intense approach.

Further studies suggest that also patients in vegetative state or older patients may benefit from early rehabilitation treatment and may also reach better outcomes [8, 25].

One of the main limitations of our study was the low response of CIQ questionnaires. On the one hand it was not possible to suggest all patient families or nursing homes to cooperate with our investigation on the other a great number of subjects moved and were lost to our follow up. As a result only 29 of 62 questionnaires could be analyzed reducing the statistical value of the parameter and making only descriptive statistics possible.

Another limitation was the major difference in trauma severity between the groups. Therefore results must be interpreted as only preliminary.

## Conclusion

After collecting all the data concerning patients who have suffered a traumatic brain injury, we conclude that the strategy of early rehabilitation may result in better short and long term outcomes. Further controlled studies are necessary to prove this approach in other specialized rehabilitation centers.
